# The brain-heart connection: Value of concurrent ECG and EEG recordings in epilepsy management

**DOI:** 10.1016/j.ebr.2024.100726

**Published:** 2024-10-30

**Authors:** Jeremy D. Slater, Selim Benbadis, Richard L. Verrier

**Affiliations:** a Stratus, Inc., Irving TX, USA; b Department of Epilepsy/Neurology, University of South Florida, Tampa General Hospital, Tampa, FL, USA; c Department of Medicine, Harvard Medical School, Beth Israel Deaconess Medical Center, Boston, MA, USA

**Keywords:** Epilepsy, Cardiac, Monitoring, Autonomic dysfunction, Epileptic heart syndrome

## Abstract

Concurrent electrocardiogram (ECG) and electroencephalogram (EEG) recording both ictally and interictally has significant value in the comprehensive management of epilepsy. This review highlights the diagnostic utility of simultaneous ECG and EEG monitoring in differentiating between epileptic and cardiac events, detecting cardiac abnormalities, and identifying autonomic dysfunction. The critical role of this combined approach to defining the mechanisms underlying cardiac morbidity and sudden cardiac death in patients with epilepsy and in guiding therapeutic interventions is underscored. The “Epileptic Heart Syndrome” is examined, illustrating how chronic epilepsy can adversely affect cardiac structure and function, leading to increased risk for interictal cardiac arrhythmias, morbidities, and mortality. The findings emphasize the need for standardized protocols for routine concurrent ECG and EEG recording in epilepsy monitoring units both ictally and interictally to ensure comprehensive patient care, improve diagnostic accuracy, and potentially reduce epilepsy-related morbidity and mortality. Future research directions are proposed to address existing gaps and to advance the technology and methodology for concurrent monitoring including wearable and computer-based monitoring systems.

## Highlights

• Chronic epilepsy can increase risk for both atrial and ventricular arrhythmias.
• Autonomic dysfunction has been implicated as a trigger of cardiac events.
• Dual ECG/EEG recording enables monitoring neurocardiac interactions.
• A systematic approach to relevant ECG and autonomic parameters is provided.
• Ictal and interictal dual ECG/EEG monitoring can enhance epilepsy management.

## 1 Introduction

Epilepsy is a neurological disorder characterized by recurrent seizures that significantly impact patients' quality of life and overall health. Traditional monitoring methods primarily focus on electroencephalogram (EEG) recordings to track brain activity during seizures. However, these methods often do not capture the full scope of physiological changes associated with seizures, particularly those affecting cardiac function.

Studies have shown that concurrent electrocardiographic (ECG) and electroencephalographic (EEG) monitoring can detect significant cardiac abnormalities during seizure, such as tachycardia, bradycardia, and asystole, which might not be apparent through EEG alone. These findings underscore the necessity of integrating ECG and EEG monitoring in routine epilepsy management to improve diagnostic accuracy and ensure comprehensive patient care.

Recent advances have highlighted the importance of incorporating ECG recordings alongside the EEG to provide a more comprehensive view of the interactions between the brain and heart during epileptic events. Concurrent ECG and EEG recording allows for the simultaneous monitoring of neurological and cardiac activities, offering critical insights into the physiological changes that occur during seizures. This combined approach is particularly valuable in identifying interictal neurocardiac dysfunction and cardiac morbidity, which can lead to sudden cardiac death (SCD).

This review aims to consolidate current knowledge on the value of concurrent ECG and EEG recording, highlighting its significance in clinical practice and proposing future research directions to improve patient outcomes. It addresses monitoring cardiac comorbidities and risk for SCD in epilepsy rather than sudden unexpected death in epilepsy (SUDEP), which by definition excludes cardiac death [1] and is generally considered to result from respiratory failure [2], [3].

## 2 Brain-Heart Neurocircuitry

**Fig. 1:**
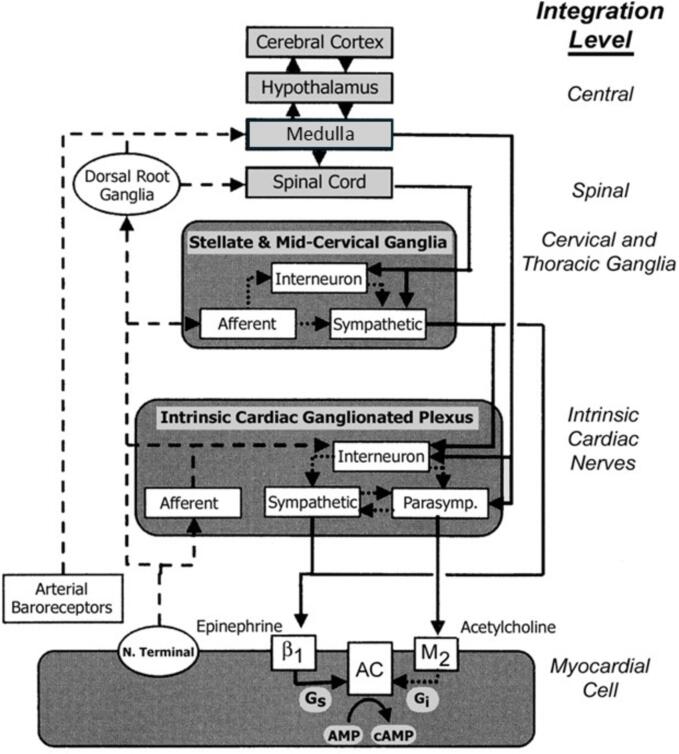
Traditional concepts of neural control of cardiac electrical activity focused on afferent tracts (dashed lines) arising from myocardial nerve terminals and reflex receptors (e.g., baroreceptors) that are integrated centrally within hypothalamic and medullary cardiostimulatory and cardioinhibitory brain centers and on central modulation of sympathetic and parasympathetic outflow (solid lines) with little intermediary processing at the level of the spinal cord and within cervical and thoracic ganglia. More recent views incorporate intricate processing within the extraspinal cervical and thoracic ganglia and within the cardiac ganglionic plexus, where interneurons are envisioned to provide new levels of noncentral integration. Release of neurotransmitters from postganglionic sympathetic neurons is believed to enhance excitation in the sino-atrial node and myocardial cells through norepinephrine binding to beta-1 receptors, which enhances adenyl cyclase (AC) activity through intermediary stimulatory G proteins (Gs). Increased parasympathetic outflow enhances postganglionic release and binding of acetylcholine to muscarinic (M2) receptors, and through coupled inhibitory G proteins (Gi), inhibits cyclic AMP production (cAMP). The latter alters electrogenesis and pacemaking activity by affecting the activity of specific membrane Na, K, and Ca channels. New levels of integration are shown superimposed on previous views and are emphasized here to highlight new possibilities for intervention. Reprinted with permission from Lathrop and Spooner [158].

The heart is a wired organ, with critical neurocardiac connections that occur at multiple levels from the brain to the intrinsic cardiac nervous system. This intricate, multilevel integration scheme is illustrated in Fig. 1. In patients with chronic epilepsy, during seizure, hyperactive engagement of the neural networks in the brain and heart leads to abnormalities of sinus and atrioventricular nodal function and atrial and ventricular tissue. This intensely activated neuronal state has the potential for triggering rapid cardiac rhythms including atrial tachycardia and fibrillation and even life-threatening ventricular arrhythmias. This tight brain/heart linkage provides a sound fundamental basis for concurrent measurement of the EEG and ECG.

## 3 Diagnostic utility

The diagnostic utility of concurrent ECG and EEG recording lies in its capacity to provide a comprehensive view of both neurological and cardiac activities during and following epileptic events, significantly enhancing the accuracy of diagnosis. This approach is particularly valuable in distinguishing epileptic from cardiac events.

Almost every EEG recording has an accompanying single-lead ECG recording, generally lead I, which typically lasts for the duration of the EEG monitoring session, whether a routine 20- to 30-min session or an extended session lasting one or more days. According to Herder [4], “The traditional use of this ECG tracing is to differentiate ECG artifact from abnormal brain activity.“ While sufficient for the detection of many cardiac abnormalities and identification of ECG artifact in the EEG, the single lead is frequently obscured by movement and/or artifact or is lost entirely.

The U.K.’s National Institute for Health and Care Excellence (NICE) guidelines [5] recommend 12-lead resting and ambulatory ECGs in epilepsy patients in cases of transient loss of consciousness > 16 sec in order to rule out vasovagal/cardiac syncope in the diagnosis of seizure and epilepsy. To our knowledge, the NICE guidelines [5] are the sole source of professional recommendations for ECG monitoring and analysis of patients with epilepsy. Petkar and colleagues [6] reported results of the Reveal in the Investigation of Syncope and Epilepsy (REVISE) study, which addressed the 13 %-42 % of misdiagnoses of epilepsy in England. An insertable loop ECG recorder (ILR) was used to confirm cardioinhibition during syncope and to provide evidence of the likely alternative diagnosis of convulsive reflex syncope, which can mimic an epileptic seizure. Patients with bradyarrhythmia or asystole were offered cardiac pacemaker implantation, and antiseizure medication (ASM) was withdrawn, with 60 % of patients responding. Other investigators provided further case studies of reliance on ambulatory ECG recorder, one-lead ECG during video monitoring, or ILR to correctly diagnose syncope vs. epilepsy [7], [8], [9], [10], [11], [12], [13], [14], [15], [16], [17].

### 3.1 Syncope and ictal cardiac asystole

Multiple case reports and case series attest to the potential of ECG monitoring to lead to accurate diagnoses of syncope and ictal cardiac asystole [7], [8], [9], [10], [11], [12], [13], [14], [15], [16], [17], [18], [19], [20], [21], [22], [23], [24], [25], [26], [27], [28], [29], [30], [31], [32], [33]. In particular, Agostini et al [25], Bestawros et al [27], Kishk et al [29], and Sowden and colleagues [33] indicated the importance of concurrent EEG-ECG monitoring in diagnosis of ictal cardiac asystole/syncope and consideration of permanent pacemaker implantation, which was employed by a number of physicians to treat ictal asystole and bradycardia [34], [35], [36].

Rocamora et al [20], Kouakam and colleagues [22], and Mehvari et al [26] underscored the potential of ECG monitoring to contribute to differential diagnosis of cardiac origin of asystole. Ficker et al [9] illustrated the importance of concurrent monitoring through a case study in which prolonged video-EEG monitoring revealed that a patient's episodes, initially presumed to be due to temporal lobe epilepsy, were cardiac asystole. This accurate diagnosis allowed for the discontinuation of unnecessary antiepileptic drugs and the insertion of a cardiac pacemaker. Venkataraman et al [12] reported on patients with intractable seizure disorders, where simultaneous scalp video EEG and ECG recordings led to the diagnosis of asystole rather than seizures. This accurate diagnosis prompted the implantation of a cardiac pacemaker, which successfully prevented further paroxysmal episodes. Mayor et al [17] further supported the diagnostic value of concurrent ECG and EEG recording in identifying cardiogenic syncope in patients previously diagnosed with epilepsy. Their study used one-lead ECG during video-EEG assessment, which allowed for detecting arrhythmias. Nandkeolyar et al [30] reported cases in which ictal asystole was documented through video-EEG-ECG monitoring, leading to the decision to implant a cardiac pacemaker.

### 3.2 Ictal cardiac arrhythmias

Concurrent ECG and EEG recording is instrumental in detecting and diagnosing cardiac arrhythmias that frequently accompany epileptic seizures, such as ventricular tachycardia (>100 beats/min) and bradycardia (<60 or < 50 beats/min) [34], [37], [38], [39], [40], [41], [42], [43], [44], [45], [46], [47], and is crucial to understanding the full spectrum of physiological changes during seizures, with significant implications for patient management and safety. Li et al [34] reported that 39 % of seizures were associated with tachycardia and 5 % with bradycardia. Monté and colleagues [48] discovered an association between ictal bradycardia and brain ischemia.

**Fig. 2:**
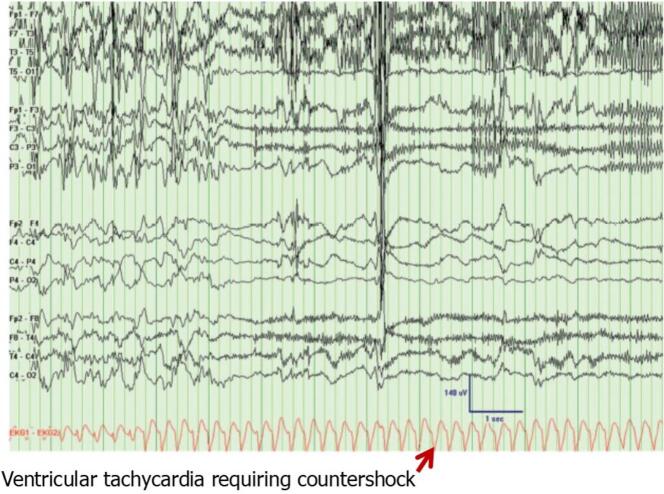
Seizure-induced malignant arrhythmia in an epilepsy monitoring unit patient. Electroencephalogram (EEG) showing the end of a right temporal lobe seizure with electrocardiogram (ECG) revealing a rapid ventricular tachycardia degenerating into pulseless ventricular fibrillation requiring countershock. Patient was a 51 year-old woman with a history of epilepsy since childhood. Reprinted with permission from the American Academy of Neurology [53].

Kendirli and colleagues [49] determined that 18 % of patients undergoing EEG testing exhibited cardiac arrhythmias. Marshall et al [50] noted that seizure-induced ventricular tachycardia could have serious consequences in patients with cardiac disease. Tigaran et al [51], [52] and Nousiainen et al [40], [41] proposed that seizure-induced ECG abnormalities could contribute to differential diagnosis of cardiac disease. ST-segment depression during seizures has been noted in ∼ 40 % of patients and should alert physicians to myocardial ischemia [51], [52]. Espinosa and colleagues [53] provided a recording of concurrent measurement of EEG and ECG in the epilepsy monitoring unit (EMU) (Fig. 2) illustrating that seizure can lead to ventricular tachycardia requiring cardioversion.

Seizure increases the prevalence of atrial fibrillation (AF), which if untreated can increase risk for stroke. Post-ictal atrial fibrillation has been documented [44], [54], [55], [56], [57], [58], [59], [60], [61], [62], [63]. AF was reported in 9.7 % of 1.4 million hospitalizations of U.S. epilepsy patients [64] compared to 2 % in the general population, a 4.9-fold increase. Likewise, Wang and colleagues [65] reported a 1.26-fold increase in atrial fibrillation incidence in patients with epilepsy enrolled in the U.K. Biobank compared to the general population.

### 3.3 Long QT syndrome

Case reports and case series across > 35 years have reported misdiagnoses of the long QT syndrome, a cardiac pathology, as epilepsy based on transient loss of consciousness; the accurate diagnosis can be confirmed by genetic testing following ECG monitoring [19], [66], [67], [68], [69], [70], [71], [72], [73], [74], [75], [76], [77], [78], [79], [80], [81], [82], [83], [84], [85], [86].

Ramos-Maqueda and colleagues [84] calculated that misdiagnosis of long QT syndrome as epilepsy is associated with a 6.92-fold increased risk of sudden cardiac arrest/SCD, with multi-year diagnostic delays. More recently, genetic studies have disclosed common channelopathies in patients with epilepsy and the long QT syndrome [81], [87], [88], [89], [90], [91], [92], [93], [94], a subject that deserves further study.

## 4 Interictal autonomic dysfunction

Interictal ECG monitoring is crucial for detecting autonomic dysfunction in patients with epilepsy based on heart rate variability (HRV) analysis [95], [96], [97], [98], [99], [100], [101]. Both Massetani et al [96] and Hamdy et al [101] highlighted the value of simultaneous ECG and EEG recording in identifying autonomic dysfunction in patients with temporal lobe epilepsy, reporting that HRV analysis revealed decreased low-frequency (LF) and high-frequency (HF) components in patients with epilepsy compared to control subjects. In addition, both Yildiz and colleagues [97] and Hamdy et al [101] reported increases in LF/HF ratio among refractory epileptic patients compared to nonepileptic control subjects. These findings suggest impaired parasympathetic nerve tone and increased sympathetic activity, an autonomic state that increases risk for cardiac arrhythmias. Hamdy and coworkers [101] also reported that root mean square of successive beat-to-beat interval differences (rMSSD), an HRV marker of parasympathetic activity, was reduced in epileptic patients.

Ponnusamy and colleagues [98] discovered significant differences between ictal HRV measures during epileptic compared to nonepileptic seizures that could aid seizure classification. Myers and coworkers [99] determined that autonomic dysfunction based on HRV measurements was particularly severe in patients with epilepsy and sodium channel mutations and called for a minimum protocol for HRV evaluation to be used in all studies involving epilepsy patients [100].

## 5 The “Epileptic Heart Syndrome”

Characterization of the “Epileptic Heart Syndrome“ has emerged from evidence in the literature reporting that chronic epilepsy can negatively impact cardiac structure and function, lead to myocardial and vascular injury, and increase risk for *peri*-ictal and interictal cardiac arrhythmias, including ventricular fibrillation [44], [102], [103], [104], [105], [106], the arrhythmia responsible for SCD [107]. Verrier et al [108], [109] hypothesized that repeated seizure-induced surges in catecholamines and hypoxemia lead to myocardial and coronary vasculature damage and result in cardiac electrical and mechanical dysfunction. Fialho and colleagues [110] surveyed the medical literature and presented evidence of myocardial injury (including myocardial ischemia and fibrosis and left ventricular stiffness) and premature cardiovascular mortality in patients with epilepsy that could be attributed to seizure-induced catecholaminergic toxicity. Population studies indicate significant increases in cardiac morbidities in patients with epilepsy, including myocardial infarction to 4.92-fold [111], ischemic heart disease to 4.18-fold [112], heart failure to 1.56-fold [113], SCD and cardiac arrest to 6.65-fold [63], atrial fibrillation to 4.9-fold [64] and ventricular tachycardia and fibrillation to 1.1-fold [114] compared to the general population [115]. Bardai and colleagues [104] reported a 2.9-fold increase in sudden cardiac arrest incidence due to ventricular fibrillation documented by automated external defibrillator ECG recordings in the Amsterdam Resuscitation Studies (ARREST). Rezk and colleagues [116] calculated the 2.25-fold increased odds of developing cardiac dysfunction in patients with epilepsy based on a systematic review and *meta*-analysis.

The potential for seizures and for some ASMs to accelerate atherosclerosis [117], [118] requires assessment with ambulatory ECG monitoring or resting 12-lead ECG recordings. Determining toxic effects of ASMs can be facilitated by ECG monitoring [119]. Both the American Epilepsy Society and the International League Against Epilepsy have recommended a standard 12-lead ECG in epilepsy patients > 60 years of age before starting lamotrigine, an ASM with sodium channel blocking properties, and in younger patients with known cardiac disease or risk factors for cardiovascular mortality [120]. Patients with epilepsy may require specialized care from a cardiologist to mitigate risk for premature mortality from ischemic heart disease conferred by active epilepsy [121], [122].

ECG monitoring also allows characterization of seizure-induced myocardial stunning and/or Takotsubo cardiomyopathy, which has been estimated to occur in ∼ 1 of 1000 in-hospital seizures, resulting in poor outcomes, including inpatient mortality (3.7 %), arrhythmia (22.7 %), and cardiac arrest (3.9 %), etc. [123].

Fialho and colleagues [124] applied echocardiography to identify various structural and functional cardiac abnormalities in patients with epilepsy. Their studies reveal that patients with chronic epilepsy exhibit enhanced electrical dispersion and subtle echocardiographic patterns that may be linked to heart failure with a preserved ejection fraction (HFpEF) phenotype. They also found that epilepsy can accelerate cardiovascular disease progression, as indicated by significant markers of atrial depolarization heterogeneity, ventricular repolarization heterogeneity, and left ventricular geometry abnormalities in patients with epilepsy [125], [126].

The pediatric population is not spared. Bartlett-Lee et al [127] investigated the prevalence of minor ECG abnormalities in children with epilepsy and concluded that these abnormalities are associated with longer epilepsy duration.

ECG parameters and ECG-based measures of autonomic tone for diagnosing the “Epileptic Heart Syndrome” are summarized in Table 1 [109].

**Table 1:** Recommended ECG Parameters in Evaluating Brain/Heart Interactions in Chronic Epilepsy.

• **Myocardial Injury and Arrhythmia Risk on Electrocardiogram** o Clinical signs and symptoms such as exercise intolerance, chest pain, irregular pulse and palpitations o Atrial and/or ventricular arrhythmias o P waves >2.5 mm tall and/or >110 ms wide may indicate atrial enlargement o P-wave heterogeneity o Q waves—indicator of prior myocardial infarction o QRS complex >150 ms wide may indicate conduction abnormalities and electrical dyssynchrony o Severe QT interval prolongation (>450 ms in men, >470 ms in women) may indicate repolarization abnormalities or antiseizure medication use o ST-segment depression or elevation o T-wave alternans ≥47 µV o T-wave heterogeneity • **Altered Autonomic Tone as Assessed by Heart Rate Variability Measures** o rMSSD <27±12 ms o LF/HF ratio >1.5-2.0 o HF <975±203 ms^2^

Note: ECG = electrocardiogram; HF = high-frequency; LF/HF = low-frequency/high-frequency HRV; rMSSD = root mean square of successive differences.

Reprinted with permission from Verrier et al 2021 [109].

## 6 Prevention of sudden cardiac death in epilepsy

SCD risk is a significant concern in the management of patients with epilepsy, particularly as it may or may not occur in association with seizure [128] and is likely due to the constellation of seizure-induced cardiac morbidities.

Enhanced QT prolongation, a widely used ECG marker of SCD risk, is characteristic of patients with epilepsy [129], [130], [131], [132], [133]. QTc ≥ 448 ms was demonstrated to predict 1.9-fold increased all-cause mortality in patients with seizure or epilepsy registered in the Mayo Clinic (Rochester MN) hospital medical records database [134]. It is noteworthy that ASMs [135] as well as seizures themselves [136] can prolong the QT interval in patients with epilepsy. In particular, oxygen desaturation during seizures increases QT prolongation by 4.3-fold [137]. Automated measurement of QT and corrected QT intervals (QTc) on the 12-lead ECG makes this index particularly accessible. Despite the availability of this straightforward, low-cost option to monitor QT prolongation, 12-lead ECG recordings upon admission to a hospital emergency department have not been standard for patients with epilepsy. The Mayo Clinic hospital medical records study found that only 57 % of > 18,000 patients diagnosed with epilepsy received a 12-lead ECG at emergency department admission [134].

T-wave alternans (TWA) has been shown in general and cardiac populations to be a marker of risk for lethal arrhythmias [138]. Strzelczyk and colleagues [139] reported post-ictal increases in TWA and heart rate along with decreases in HRV. Verrier and coworkers [140] discovered that interictal TWA levels in patients with epilepsy were elevated to the same degree as in high-risk patients with ventricular tachycardia following ST-segment elevation myocardial infarction. The group later reported that vagus nerve stimulation, an approved treatment to reduce seizure, also significantly reduces TWA [141]. More recently, they found in newly diagnosed epilepsy patients that interictal TWA levels are similar to normal individuals but in patients with drug-resistant epilepsy, interictal TWA levels are significantly increased and register a high degree of risk [142].

## 7 Seizure detection and forecasting

Investigators have reported that ECG monitoring enhances seizure detection and forecasting. Significant progress has been made although these goals remain elusive [143].

Greene et al [144] reported that the combination of EEG and ECG improves the accuracy of seizure detection in neonates. Olmi et al [145] described automatic detection systems that integrate EEG, ECG, and video recordings in neonatal intensive care units (NICUs) for monitoring epileptic seizures. Jeppesen et al [146], [147] described a seizure detection algorithm based on changing HRV indicators using a standard wearable device. Mporas et al [148] and Zhang and colleagues [149] proposed advances in computer-based monitoring systems integrating EEG and ECG signals for improved seizure detection based on artificial intelligence.

Cousyn and colleagues [150] found that HRV features can identify a preictal state with a median area under the receiver-operating characteristic curve of 0.75. Ghaempour and coworkers [151] described a wearable ECG monitoring system that can detect seizure with > 98 % accuracy and predict seizure with 1–2 min lead time with > 94 % accuracy based on artificial intelligence. In a pilot study, Pang et al [152] provided evidence that the magnitude of T-wave heterogeneity (TWH) exhibits a crescendo at 30 min prior to seizure without heart rate increases > 2 beats/min until 10 min prior to seizure. Acute TWH elevations may predict impending generalized tonic-clonic seizures (GTCS) and may discriminate patients with GTCS from those with behaviorally similar psychogenic nonepileptic seizures.

## 8 Recommendations for routine clinical practice

The broader implications for clinical management include developing more holistic and practical treatment plans to address neurological and cardiac issues. Simultaneous EEG monitoring with either a 3-lead wireless ECG patch or 6-lead wired ambulatory ECG recorder has been recommended by numerous investigators, who emphasized the need for standardized protocols to ensure consistent and effective use of concurrent ECG and EEG monitoring [4], [8], [12], [14], [19], [25], [26], [29], [30], [33], [34], [40], [41], [49], [74], [76], [144], [153], [154], [155]. A survey of patient monitoring in Canadian EMUs disclosed that only 65 % employed continuous ECG monitoring [156]. Sowden and colleagues [33] and Verrier et al [155] in particular called for the development of guidelines for combined ECG-EEG monitoring of patients with epilepsy. The latter stated: “Standard 12-lead ECGs should be obtained at baseline in patients with newly diagnosed or suspected epilepsy and at regular intervals to provide screening for cardiac pathologic conditions; full cardiac evaluations should be considered in patients with chronic drug-resistant epilepsy and concurrent cardiovascular risk factors.”

Overall, concurrent ECG and EEG recording has significant implications for the clinical management of epilepsy. It enables the detection of both neurological and cardiac abnormalities and informs more holistic treatment strategies.

## 9 Future directions

Future research directions should integrate ECG and EEG recording for epilepsy management and focus on developing guidelines, addressing gaps in the literature, refining methodologies, and exploring technological advances.

**Fig. 3:**
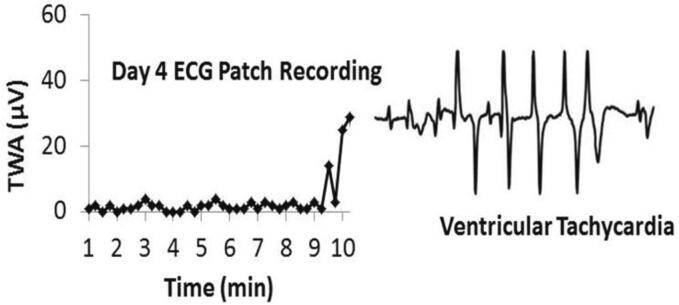
Crescendo in T-wave alternans (TWA) level heralded onset of ventricular tachycardia in a patient with chronic epilepsy. This event occurred on the 4th day of the ECG patch recording and thus the arrhythmia was not recorded by the 24-hour Holter monitor. Reprinted with permission from the American Academy of Neurology [142].

**Fig. 4:**
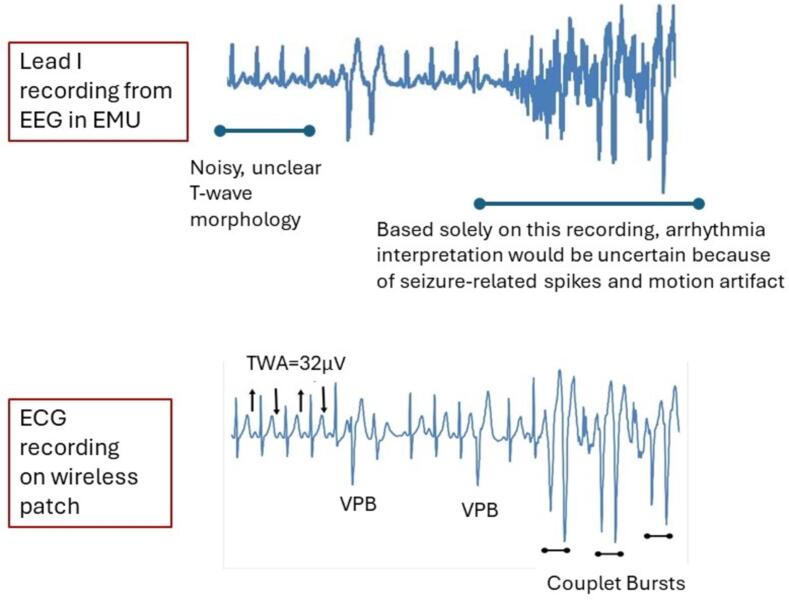
Concurrent recordings of standard lead I wired electrocardiogram (ECG) in association with the electroencephalogram (EEG) recording and wireless ECG patch in an epilepsy monitoring unit (EMU) patient experiencing ventricular premature beats (VPB). The upper tracing illustrates a relatively noisy reading typically associated with the artifacts from body movements of lead wires. The lower tracing illustrates higher quality tracings free of artifacts. The presence and morphology of ECG beats can be readily observed. TWA = T-wave alternans.

Studies on the long-term effects of epilepsy on cardiac health and the mechanisms underlying interictal cardiac dysfunction will be facilitated by improving the quality of ECG monitoring. Wireless ECG patch recordings are superior to conventional wired ambulatory or EMU system monitoring (Fig. 3, Fig. 4). Advances in wearable and computer-based monitoring systems hold great potential for revolutionizing epilepsy care, providing continuous, real-time monitoring that can enhance the ability to detect seizures and cardiac abnormalities.

## 10 Conclusions

The integration of concurrent ECG and EEG recording in epilepsy management offers significant benefits, providing a comprehensive approach to monitoring this complex neurological disorder. This review highlights the critical roles of combined monitoring in enhancing diagnostic accuracy and detecting cardiac arrhythmias and comorbidities and autonomic dysfunction. The “Epileptic Heart Syndrome” underscores the need for routine cardiovascular evaluation in epilepsy patients to identify and manage cardiac risk.

The concurrent use of ECG and EEG recordings is essential for comprehensive epilepsy management. It can enhance diagnostic accuracy and inform therapeutic decisions. By continuing to advance research and clinical practices in this area, we can better understand and manage the complex interactions between the brain and heart in epilepsy, ultimately reducing the burden of this disorder and improving the lives of patients. Integrating these monitoring techniques elevates patient care and sets a new standard for the holistic management of epilepsy, paving the way for future innovations and improvements in the field. Ultimately, improved cardiac risk assessment can lead to an effective strategy for management of patients with epilepsy, which will be the subject of a review by Pang and coworkers in this special issue [157].

## CRediT authorship contribution statement

**Jeremy D. Slater:** Writing – original draft, Conceptualization. **Selim Benbadis:** Writing – review & editing. **Richard L. Verrier:** Writing – review & editing.

## Declaration of competing interest

The authors declare the following financial interests/personal relationships which may be considered as potential competing interests: Jeremy Slater is CMO of Stratus, Inc., and reports financial support and administrative support provided by Stratus including employment, equity or stocks, and non-financial support. He owns stock in Zeto, Inc., a manufacturer of a dry electrode EEG headset. Selim Benbadis is National Medical Director of Stratus. Richard Verrier is a member of the Medical Advisory Board of Stratus and has received lecture honoraria from Stratus and from UCB.
